# The newly identified *grdA* gene confers high-level plazomicin resistance in *Salmonella enterica* serovars

**DOI:** 10.1128/aac.01606-24

**Published:** 2025-04-03

**Authors:** Ahmed F. Hikal, Shaohua Zhao, Steven Foley, Ashraf A. Khan

**Affiliations:** 1 Division of Microbiology, National Center for Toxicological Research, U.S. Food and Drug Administration 4136, Jefferson, Arkansas, USA; 2 Office of Applied Science, Center for Veterinary Medicine, U.S. Food and Drug Administration, Laurel, Maryland, USA; University of Fribourg, Fribourg, Switzerland

**Keywords:** *grdA*, plazomicin resistance, *Salmonella enterica*

## Abstract

Plazomicin, a next-generation aminoglycoside, was recently approved by the U.S. Food and Drug Administration for the treatment of multidrug-resistant complicated urinary tract infections. It is highly effective against most aminoglycoside-modifying enzymes (AMEs). Here, we report that *Salmonella enterica* strains containing the newly identified gentamicin resistance gene (*grdA*) are highly resistant to plazomicin. Heterologous expression of *grdA* in *Escherichia coli* Δ*tolC* resulted in plazomicin resistance with minimum inhibitory concentration (MIC) > 256 µg/mL. These findings reveal that GrdA confers significantly higher resistance to plazomicin than the previously known plazomicin-resistant AMEs AA (2)-Ia and APH (2″)-Iva.

## INTRODUCTION

The extensive use of antibiotics to treat bacterial infections has driven the rise of antimicrobial resistance, which is now a global health crisis. Aminoglycosides, naturally occurring antibiotics, have been used for decades to treat infections caused by gram-positive and -negative bacteria, as well as atypical mycobacteria (1). These antibiotics exert their bactericidal effect by binding to bacterial ribosomes, leading to the inhibition of protein synthesis (2). However, the emergence of aminoglycoside-modifying enzymes (AMEs) has necessitated the development of a new generation of aminoglycosides to combat multidrug-resistant (MDR) pathogens.

**Fig 1:**
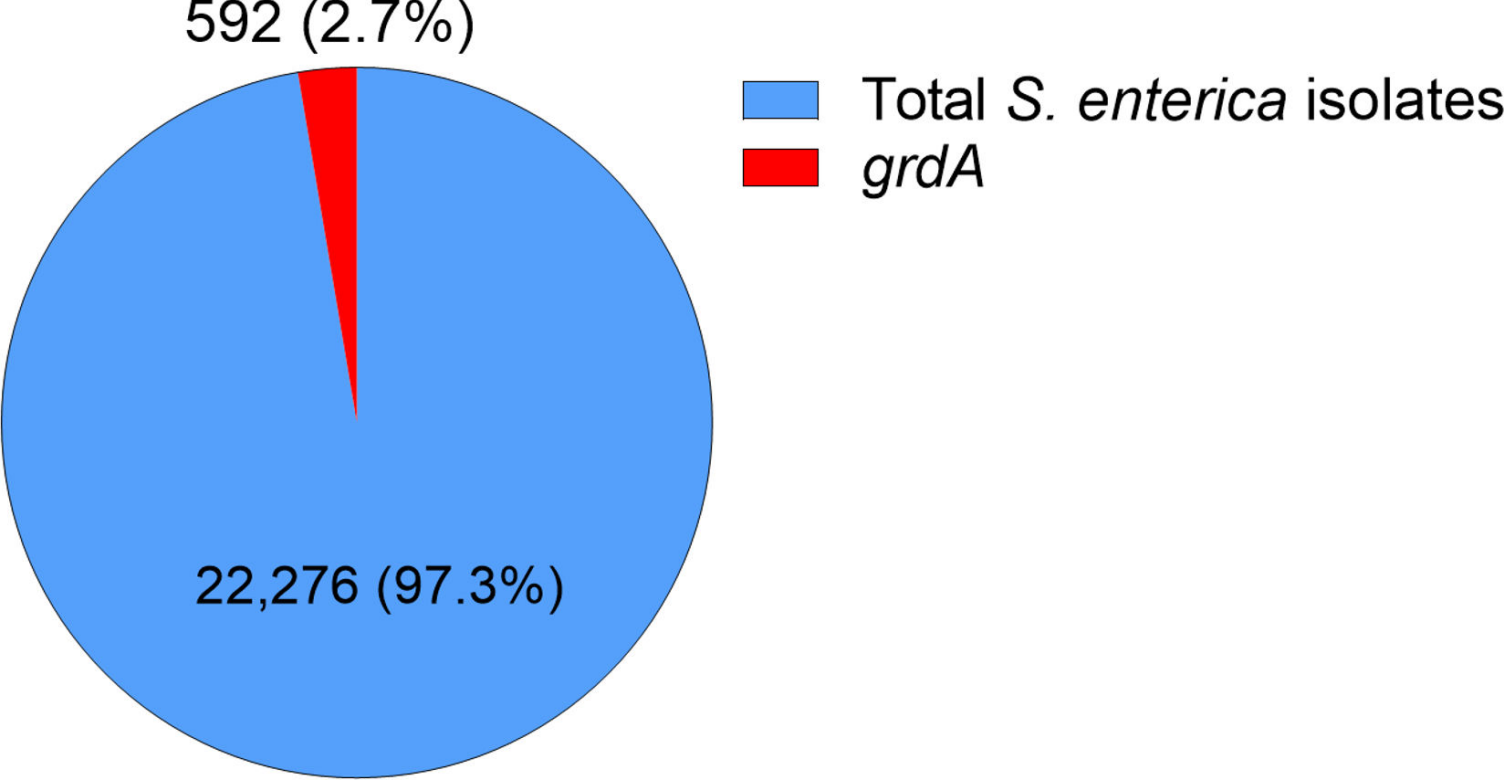
Overview of the occurrence of the *grdA* gene found across 22,276 *S*. *enterica* isolates irrespective of location on chromosomes or plasmids.

A recently identified gentamicin resistance gene, *grdA*, was found in *Salmonella enterica* isolates and confers resistance to gentamicin (3). This gene was located on a mobile genetic element that can horizontally transfer between bacteria, although the exact molecular mechanism behind this resistance remains unclear (3). The *grdA* gene encodes a protein containing a Walker A motif or P-loop G-X-X-X-X-G-K-T (X is any amino acid) common in ATP-hydrolyzing enzymes (ATPases), suggesting it may use ATP to modify gentamicin. The metadata analysis of 22,276 sequenced *S. enterica* isolates in the National Center for Biotechnology Information database reveals that 2.7% (592) carries the *grdA* gene (Fig. 1), with the majority of these isolates originating from turkey and chicken meat. In response to the rising threat of MDR bacteria, the U.S. Food and Drug Administration recently approved plazomicin, a new semisynthetic aminoglycoside designed to evade modification by many AMEs. To date, only two AMEs have been shown to confer resistance to plazomicin: aminoglycoside acetyltransferase AAC (2′)-Ia and aminoglycoside phosphotransferase APH (2″)-Iva, along with the 16S rRNA ribosomal methyltransferases (4). Additionally, the crystal structure of GrdA remains unavailable. However, the three-dimensional structure prediction of GrdA using AlphaFold (5) reveals that it consists of six alpha helices and five beta sheets. Here, we report for the first time that GrdA also confers high resistance to plazomicin.

**Fig 2:**
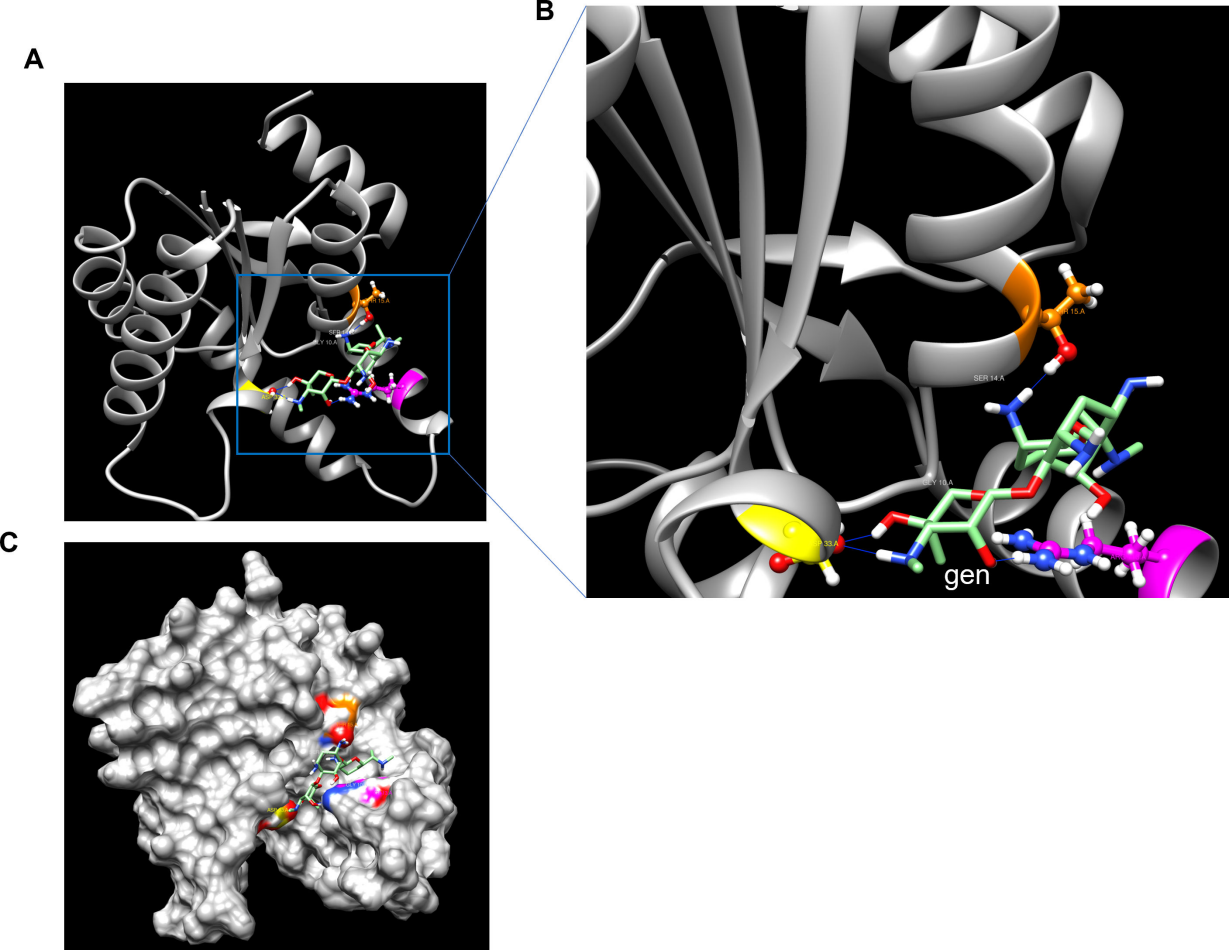
Prediction of binding of gentamicin to the GrdA monomer. A. Overall view of the GrdA monomer binding to gentamicin. B. A close-up view highlighting the key residues involved in gentamicin binding. C. GrdA surface showing the gentamicin binding pocket.

**Fig 3:**
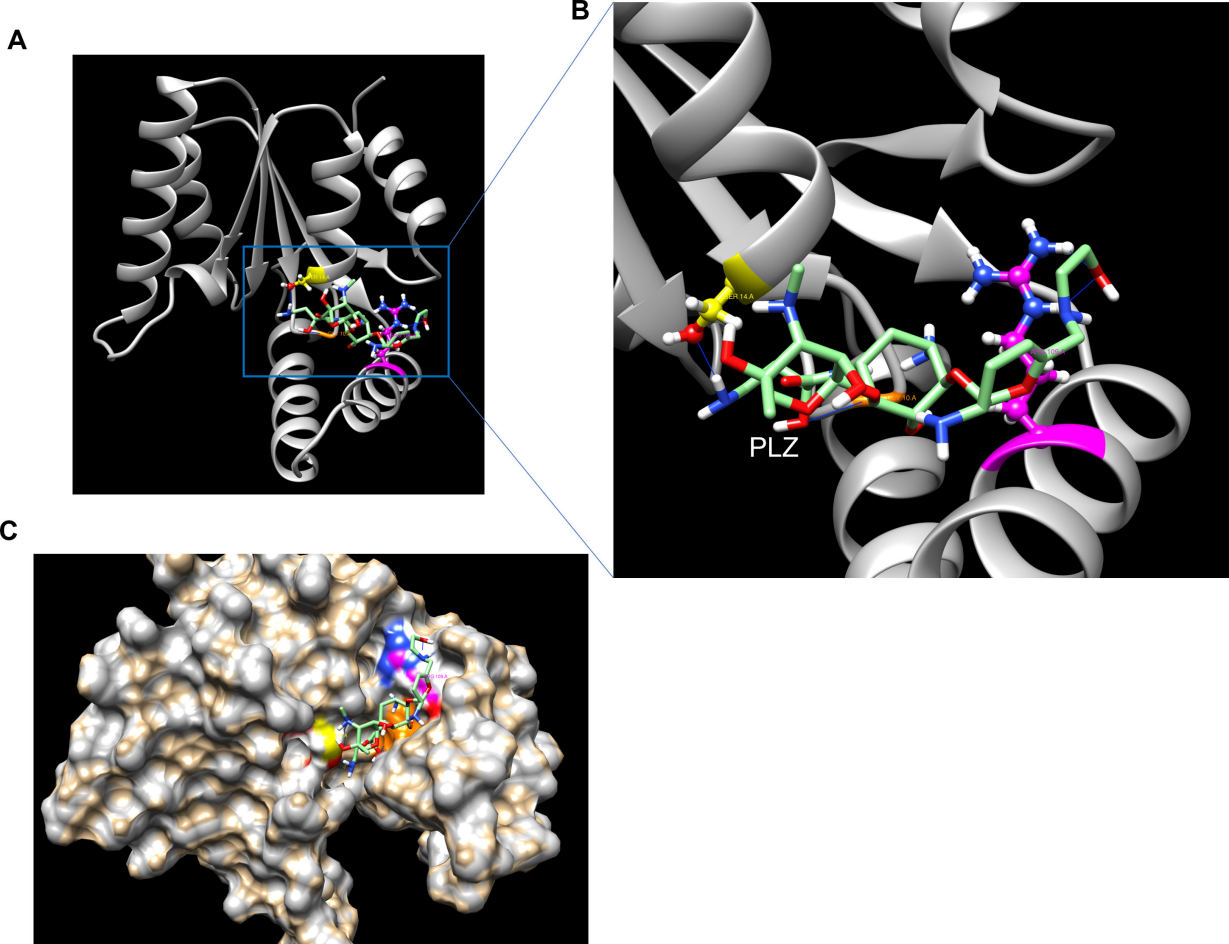
Molecular docking output of plazomicin to the GrdA monomer. A. An overview of plazomicin binding to the GrdA monomer. B. A detailed view of the amino acid residues interacting with plazomicin. C. A view of the binding pocket of GrdA.

**Fig 4:**
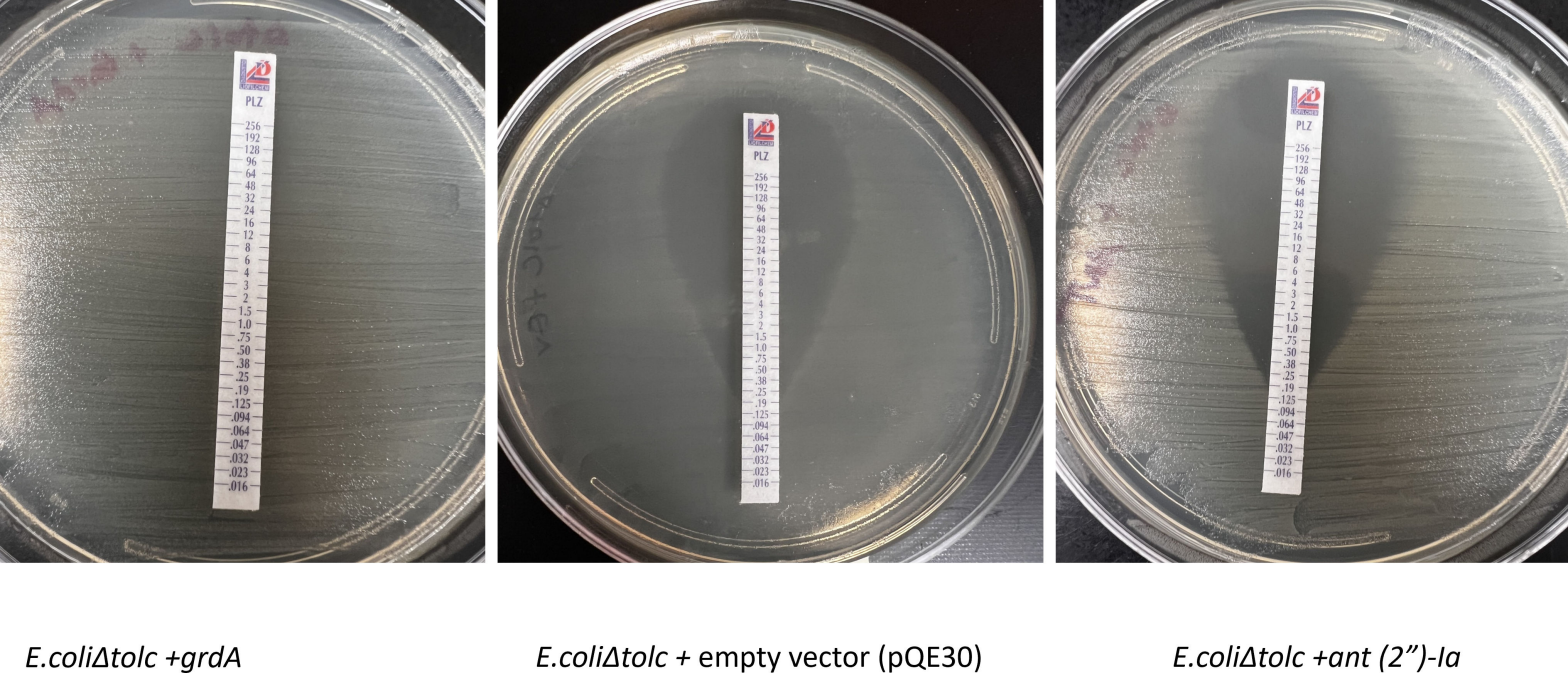
Susceptibility of *E. coli* EcM.2.1. Δ*tolC* expressing *grdA*, *ant (2″)-Ia*, or the empty vector to plazomicin using E-test.

In this study, we aimed to predict the GrdA binding sites for gentamicin and plazomicin and evaluate the susceptibility of *S. enterica* strains harboring *grdA* to plazomicin using the E-test method. Molecular docking of GrdA with gentamicin using UCSF ChimeraX (6) indicates that gentamicin binds to GrdA via four hydrogen bonds: one with ARG 113, THR 33, and two interactions with ASP 33 residues (Fig. 2A and B). Similarly, plazomicin binds to the GrdA enzyme via four hydrogen bonds, but with different amino acids: two hydrogen bonds with ARG109, one hydrogen bond with GLY10 and SER14 (Fig. 3A and B). As expected, both drugs interact with GrdA in the same binding pocket (Fig. 2C and 3C). We also assessed the activity of plazomicin in *Escherichia coli* ΔtolC expressing *grdA*. Additionally, we tested the susceptibility of *S. enterica* serovars (isolated from retail meat) harboring *grdA* and other known plazomicin-susceptible AMEs (Table 1). All strains carrying *grdA* or genes encoding AMEs (Table 1) are resistant to gentamicin; however, *S. enterica* harboring *grdA* showed minimal inhibitory concentrations (MICs) above the most recent plazomicin breakpoints (2–4 µg/mL) for *Enterobacteriaceae*, as established by the Clinical Laboratory Standards Institute (7). In contrast, *S. enterica* strains lacking *aac(2″)-Ia*, *aph (2″)-Iva*, or *grdA* remained susceptible to plazomicin with MICs < 2 µg/mL. The variation of plazomicin resistance among *grdA*-positive *S. enterica* may be due to the synergy between GrdA and AMEs or differences in GrdA expression levels (Table 1). Remarkably, *S. enterica* and *E. coli* carrying the *grdA* gene are sensitive to kanamycin, tobramycin, and amikacin in contrast to other strains harboring genes encoding AMEs. This suggests that *grdA* confers resistance exclusively to gentamicin and plazomicin. To further confirm the role of GrdA in plazomicin resistance, the *grdA* gene from SL-5 was amplified using primer pair: P-33 (5′- gtatctGAGCTCATGATCATTATTATCAACGGCCCACTG-3′) and P-34 (5′- gtataaAAGCTTTCATTCAACCCCCAGCCGC-3′), which contain *Sac*I and *Hind*III restriction sites, respectively. The PCR product was cloned into the *Sac*I and *Hind*III restriction sites of the plasmid pQE30 downstream of a T5-inducible promoter and transformed into *E. coli* EcM.2.1Δ*tolC* to rule out the possibility of plazomicin efflux via TolC. As a negative control, we transformed the empty vector (pQE30) into the same strain. Additionally, we cloned the plazomicin-susceptible aminoglycoside nucleotidyltransferase-encoding gene *ant (2″)-Ia* into *E. coli* EcM.2.1Δ*tolC* as a second negative control for the susceptibility testing. The *ant (2″)-Ia* gene was amplified from SL-26 using the primer pairs P-83 (5′-CAGTCAGAGCTCATGGACACAACGCAGGTCAC-3′) and P-84 (5′- TCAGTCAAGCTTCGTCGGCTTGGACGAATTGTTAG-3′), which also contain *Sac*I and *Hind*III, respectively. The PCR product was cloned into the *Sac*I and *Hind*III sites of plasmid pQE30, and the newly generated plasmid was transformed into the Δ*tolC* strain. Expression of *grdA* in *E. coli* EcM.2.1Δ*tolC* resulted in high-level plazomicin resistance (MIC > 256 µg/mL), which is 6- and 9-fold higher than the MICs conferred by *aac(2′)-Ia* and *aph(2″)-Iva*, respectively (Fig. 4). In contrast, strains harboring the empty vector or *ant(2″)-Ia* remained susceptible to plazomicin (MIC < 0.5 µg/mL) (Fig. 4).

**TABLE 1:** MICs (µg/mL) of aminoglycosides against *S. enterica* and *E. coli* Δ*tolC* containing *grdA* and/or genes encoding AMEs^*a*^

Strain	Serovar	Name	AMEs*/grdA*	PLZ	GM	K	TOB	AMK
*S. enterica* ATCC 13076	Enteritidis	WT	Negative	0.75	0.75	2	1	1.5
*S. enterica* N29351	Bredeney	SL-1	*grdA*	32	128	3	2	2
*S. enterica* N32755	Senftenberg	SL-4	*grdA, aac(6')-Ib4, aadA1*	>256	>256	96	8	3
*S. enterica* N32779	Senftenberg	SL-5	*grdA*	32	128	3	1.5	3
*S. enterica* N46827	Albany	SL-9	*grdA*	12	48	3	1.5	6
*S. enterica* N29362	Heidelberg	SL-16	*aac (3)-IId, aadA1*	1	128	12	8	3
*S. enterica* N50443	Dublin	SL-26	*ant(2″)-Ia, aph(3″)-Ib, aph (6)-Id*	2	96	192	64	6
*E. coli EcM.2.1*. Δ*tolC* + pAH1	NA^*b*^	NA	*grdA*	>256	>256	0.75	0.125	0.5
*E. coli EcM.2.1*. Δ*tolC* + pAH9	NA	NA	*ant(2″)-Ia*	0.5	32	96	24	0.75
*E. coli EcM.2.1*. Δ*tolC* + EV	NA	NA	Negative	0.5	0.5	0.75	0.03	0.5

^*a*^ PLZ: plazomicin; K: kanamycin; AMK: amikacin; GM: gentamicin; TOB: tobramycin.

^*b*^ NA, not applicable.

**Fig 5:**
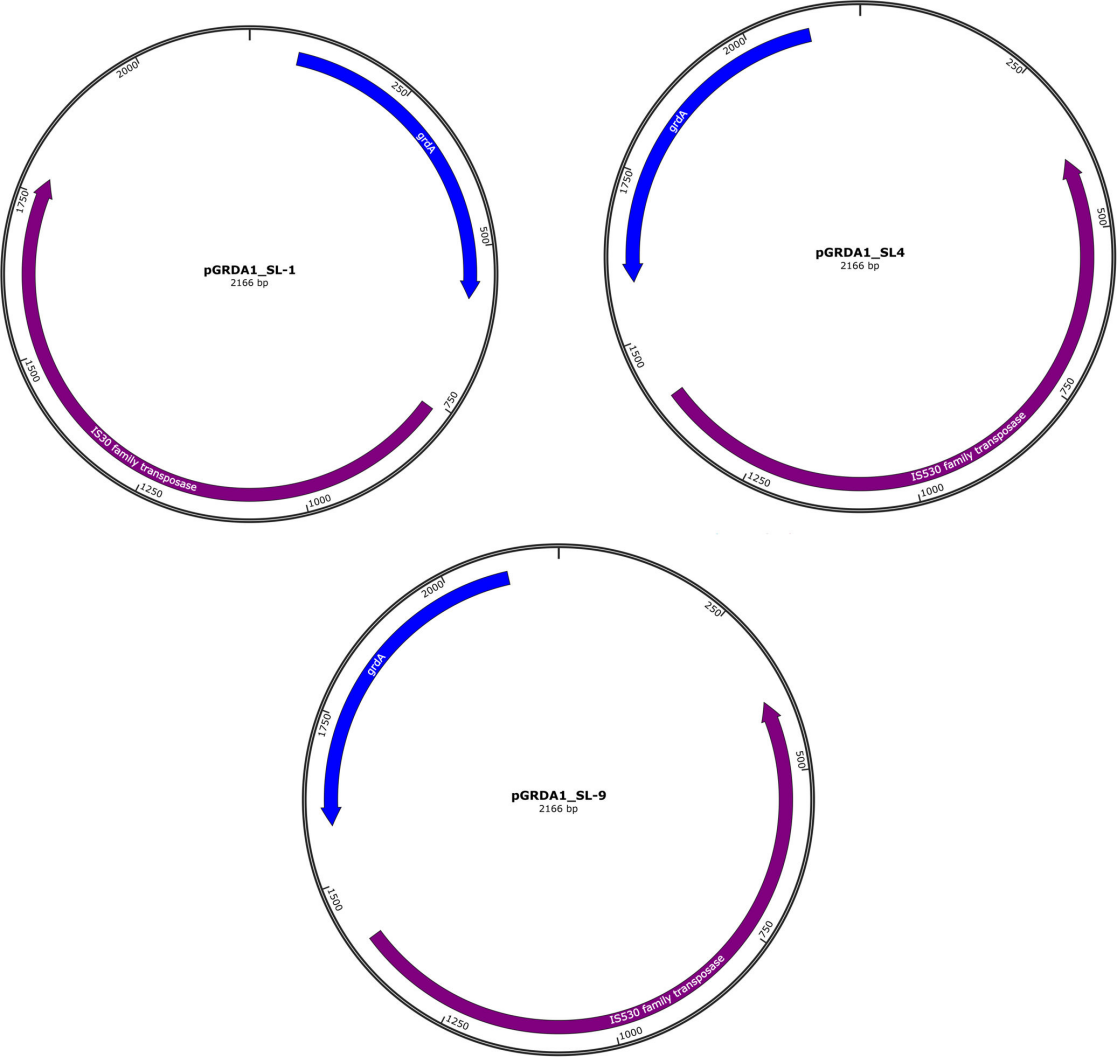
Map of the *grdA*-containing plasmid (pGRDA1) identified in SL-1, SL4, and SL-9 isolates. The plasmid was predicted by PlasmidHunter and generated in SnapGene software.

Interestingly, the level of GrdA contribution to plazomicin resistance is comparable to that conferred by the 16S rRNA methyltransferases (4), which inhibit aminoglycoside binding through modifying the 16S ribosomal RNA (8). Amino acid sequence alignment of GrdA with the most common 16S rRNA methyltransferases, ArmA, RmtA, NpmA, and NpmB, revealed less than 30% identity (Table 2), suggesting that GrdA is unlikely to be a 16S rRNA methyltransferase. Although a previous study showed that *aac(2′)-Ia* and *aph (2″)-Iva* confer resistance to plazomicin (4), their distribution is limited to the opportunistic pathogens *Providencia stuartii* and *Enterococcus* species, respectively. The *aac(2′)-Ia* gene is detected only in the chromosome of *P. stuartii* and suggested to be involved mainly in bacterial cell function (9, 10), while *aph (2″)-Iva* is restricted to *enterococci*, with no evidence of interspecies transfer (11). Notably, plazomicin is not recommended for treating *Enterococcus* infections due to intrinsic resistance conferred by the 16S rRNA methyltransferase EfmM (11, 12). However, *grdA* is mainly located on a small plasmid in *S. enterica* and is likely to transfer to other bacterial species, as it is flanked by the IS*30* family transposases (Fig. 5), raising concerns about the spread of plazomicin resistance within the *Enterobacteriaceae* family. Surprisingly, *grdA* is located on the chromosome in the SL-5 isolate and is not flanked by IS30 family transposases, which may indicate the potential for *grdA* integration into the chromosome. In summary, this is the first report of plazomicin resistance conferred by the newly identified gentamicin resistance gene (*grdA*) in *S. enterica*. Our findings underscore the need to further explore GrdA’s mechanism of resistance and develop alternative strategies for treating infections caused by *grdA*-positive *Enterobacteriaceae*.

**TABLE 2:** Sequence identities for GrdA against most common 16S rRNA methyltransferases

Protein	Identity (%)
ArmA	17.31
RmtB	19.11
NpmA	15.44
NpmB	25.00

## References

[1] Doi Y, Wachino JI, Arakawa Y. 2016. Aminoglycoside resistance: the emergence of acquired 16s ribosomal rna methyltransferases. Infect Dis Clin North Am 30:523–537. doi:10.1016/j.idc.2016.02.011 27208771 PMC4878400

[2] Makii JM, Delic J, Traeger J. 2022. Edited by H. Prabhakar. Essentials of evidence-based practice of neuroanesthesia and Neurocritical Care, p 77–88. Academic Press.

[3] Kim H-SL, Rodriguez RD, Morris SK, Zhao S, Donato JJ. 2020. Identification of a novel plasmid-borne gentamicin resistance gene in nontyphoidal Salmonella isolated from retail turkey. Antimicrob Agents Chemother 64:e00867-20. doi:10.1128/AAC.00867-20 32816720 PMC7577141

[4] Cox G, Ejim L, Stogios PJ, Koteva K, Bordeleau E, Evdokimova E, Sieron AO, Savchenko A, Serio AW, Krause KM, Wright GD. 2018. Plazomicin retains antibiotic activity against most aminoglycoside modifying enzymes. ACS Infect Dis 4:980–987. doi:10.1021/acsinfecdis.8b00001 29634241 PMC6167752

[5] Jumper J, Evans R, Pritzel A, Green T, Figurnov M, Ronneberger O, Tunyasuvunakool K, Bates R, Žídek A, Potapenko A, et al.. 2021. Highly accurate protein structure prediction with AlphaFold. Nature New Biol 596:583–589. doi:10.1038/s41586-021-03819-2 PMC8371605 34265844

[6] Meng EC, Goddard TD, Pettersen EF, Couch GS, Pearson ZJ, Morris JH, Ferrin TE. 2023. UCSF ChimeraX: tools for structure building and analysis. Protein Sci 32:e4792. doi:10.1002/pro.4792 37774136 PMC10588335

[7] CLSI. 2023. Performance standards for antimicrobial susceptibility testing. CLSI Supplement M100. Clinical and Laboratory Standards Institute, Wayne, PA.

[8] Lioy VS, Goussard S, Guerineau V, Yoon E-J, Courvalin P, Galimand M, Grillot-Courvalin C. 2014. Aminoglycoside resistance 16S rRNA methyltransferases block endogenous methylation, affect translation efficiency and fitness of the host. RNA 20:382–391. doi:10.1261/rna.042572.113 24398977 PMC3923132

[9] Payie KG, Rather PN, Clarke AJ. 1995. Contribution of gentamicin 2’-N-acetyltransferase to the O acetylation of peptidoglycan in Providencia stuartii. J Bacteriol 177:4303–4310. doi:10.1128/jb.177.15.4303-4310.1995 7635816 PMC177177

[10] Macinga DR, Rather PN. 1999. The chromosomal 2’-N-acetyltransferase of Providencia stuartii: physiological functions and genetic regulation. Front Biosci 4:D132–D140. doi:10.2741/macinga 9924143

[11] Tsai SF, Zervos MJ, Clewell DB, Donabedian SM, Sahm DF, Chow JW. 1998. A new high-level gentamicin resistance gene, aph(2’’)-Id, in Enterococcus spp. Antimicrob Agents Chemother 42:1229–1232. doi:10.1128/AAC.42.5.1229 9593155 PMC105784

[12] Gilmore MS, Clewell DB, Ike Y, Shankar N, eds. 2014. Enterococci: From Commensals to Leading Causes of Drug Resistant Infection. Eye and Ear Infirmary, Massachusetts. 24649510

